# Novel targeted therapies in chronic myeloid leukemia

**DOI:** 10.1016/j.pscia.2024.100052

**Published:** 2024-09-07

**Authors:** Muhammad Sameer Ashaq, Qian Zhou, Zhuoran Li, Baobing Zhao

**Affiliations:** a Key Lab of Chemical Biology (MOE), School of Pharmaceutical Sciences, Cheeloo College of Medicine, Shandong University, Jinan, Shandong, 250012, China; b NMPA Key Laboratory for Technology Research and Evaluation of Drug Products, School of Pharmaceutical Sciences, Cheeloo College of Medicine, Shandong University, Jinan, Shandong, 250012, China; c Department of Pharmacology, School of Pharmaceutical Sciences, Cheeloo College of Medicine, Shandong University, Jinan, Shandong, 250012, China

**Keywords:** Chronic myeloid leukemia, Stem cells, BCR-ABL1, Tyrosine kinase inhibitors, Targeted therapy

## Abstract

Chronic myeloid leukemia (CML) is the chronic proliferation of myeloid-lineage cells in hematopoietic stem cells driven by the BCR-ABL1 fusion oncoprotein. The development of tyrosine kinase inhibitors (TKIs) has revolutionized CML treatment; however, resistance and intolerance to these drugs remain key challenges. CML stem cells (CMLSCs) are the root cause of CML relapse and resistance to TKIs. This review discusses novel targeted therapeutic options targeting CMLSCs to address the abovementioned challenges. Numerous novel TKIs, such as flumatinib, vodobatinib, and olverembatinib, have shown remarkable potential against BCR-ABL1, but few, including AT9283, MK0457, and DCC-2036, are still undergoing clinical trials. Targeting CMLSCs is a fundamental therapeutic approach for the treatment of CML progression, relapse, and TKI resistance. In this review, novel agents targeting core signaling pathways and novel molecular targets in CMLSCs are highlighted. Currently, multiple approaches, such as targeting epigenetic modifications or microRNAs and altering metabolism in leukemic cells, have shown desirable effects in treating CML. Immunotherapy, autophagy inhibitors, and protein synthesis inhibitors are novel and effective therapies for the treatment of CML. Although various therapeutic strategies have provided exceptional results in the treatment of CML, the challenges of TKI resistance and CML remission or relapse remain. Therefore, current therapeutic approaches and targeted therapies have practical and clinical implications for achieving desirable outcomes.

## Abbreviation list

ABL: Abelson murine leukemia virus
AC: Accelerated phase
BCP: Blast crisis phase
BCR: Breakpoint cluster region
BET: Bromodomain and Extra-Terminal
BMM: Bone marrow microenvironment
CML: Chronic Myeloid Leukemia
CP: Chronic phase
EZH2: Enhancer of zester homolog 2
Gli: Glioma-associated oncogene
GMP: Granulocyte-macrophage progenitors
HDAC: Histone Deacetylase
Hh: Hedgehog
HIF: Hypoxia-inducible factor
HSCs: Hematopoietic stem cells
IL1RAP: Interleukin-1 receptor accessory protein
IM: Imatinib
JAK2: Janus kinase
KD: Kinase domain
LSCs: Leukemic stem cells
MAPK: Mitogen-activated protein kinase
MPL: Thrombopoietin receptor
PD: Patient-derived
Ph: Philadelphia chromosome
PP2A: Protein Phosphatase 2A
PPARγ: Peroxisome proliferator-activated receptor gamma
PRMT: Protein Arginine Methyltransferase
PTCH: Patched Hh receptor
ROS: Reactive oxygen species
SMO: Smoothened
STAT: Signal transducer and activator of transcription
TFR: Treatment free-remission
TKI: Tyrosine kinase inhibitor

## 1 Introduction

Chronic myeloid leukemia (CML) is a myeloproliferative neoplasm characterized by uncontrolled myeloid cell proliferation at all stages of differentiation [1]. It is mainly characterized by the Philadelphia (Ph) chromosome, a reciprocal translocation between chromosomes 9 and 22 (t9; 22) that results in the fusion of the breakpoint cluster region (BCR) and Abelson murine leukemia viral oncogene homolog (ABL) genes [2]. This fusion usually encodes a BCR-ABL1 protein of 210 ​kDa (BCR-ABL1^p210^), which serves as a “fingerprint” that differentiates CML from other types of leukemia [3]. Other variable BCR-ABL1 isoforms based on the different breakpoints in *BCR*, such as BCR-ABL1^p190^ and BCR-ABL1^p230^, are rarely present in CML [4].

The conventional treatment for CML, before the development of tyrosine kinase inhibitor (TKI) therapy targeting BCR-ABL1, included splenic radiotherapy, nonspecific chemotherapeutics (interferon-alpha, hydroxyurea), and allogeneic stem cell transplantation [5,6]. Although TKIs have significantly improved the prognosis of patients with CML, the treatment of patients with “blast-phase (BP)” CML remains a challenge. Acquired resistance to TKIs caused by mutations in BCR-ABL1 accounts for disease relapse and progression [7].

Residual leukemia stem cells (LSCs) are another cause of CML relapse [8]. CMLSCs, which originate from specific genomic alterations in the CD34^+^CD38^−^ subset of hematopoietic stem cells (HSCs), possess the characteristics of quiescence and self-renewal as in normal HSCs. They are thought to be an important source of TKI resistance, including both primary and acquired resistance [7,9]. CMLSCs share similar cell surface phenotypes with HSCs, such as Lin^−^CD34^+^CD38^−^, but also possess specific surface antigens, including Siglec-3 (CD33), CD36, CD44, CD47, CD52, CD25, CD26, and IL-1RAP [10,11]. Targeting CMLSCs is a critical strategy for CML treatment.

In this review, we highlight novel therapies to address TKI resistance and novel targeted therapies targeting CMLSCs to avoid relapse and remission.

## 2 Novel TKIs against BCR-ABL1 kinase domain mutations

Crystallographic studies have identified several critical residues within the ATP binding site that form direct hydrogen bonds or produce van der Waals interactions with imatinib (IM) [12]. The spectrum of mutations identified in patients with TKI-resistant CML is largely centered around the phosphate-binding loop (P loop; positions M244, G250, Q252, Y253, and E255), gatekeeper residues (T315 and F317), SH2 contact sites, the C-lobe (M351 and F359), and the activation loop (H396) [13]. These mutations produce resistance or intolerance to TKIs, which leads to constant iterations of its renewal. Several TKIs have been approved for the treatment of CML in the USA, including first-generation (imatinib), second generation (2G) (dasatinib, nilotinib, radotinib, and bosutinib), and third-generation (3G) (ponatinib) TKIs [14]. Recently, asciminib, (4G TKI) has shown remarkable potential against BCR-ABL1 kinase domain mutations and it is now commercially available [15]. The abovementioned TKIs have been reviewed in detail elsewhere; therefore, only novel TKIs are discussed in this review (Table 1).

**Table 1:** Current Clinical Trials of Novel TKIs for CML therapy.

Agent	Target	Objective	CML Phase	ClinicalTrials.gov Number	Clinical Trial Phase	Ref.
Flumatinib	c-ABL, PDGFRβ, and c-KIT	Safety and efficacy of flumatinib as first-line therapy	Chronic phase	NCT04677439, NCT04933526, NCT05353205	IV	[16]
Olverembatinib (HQP1351)	BCR-ABL1	Safety, efficacy, and pharmacokinetics of olverembatinib in patients with 2G TKI failure or intolerance	Chronic and blast phase	NCT05376852, NCT05311943, NCT03883100, NCT03883087, NCT0412668, NCT04260022	II/III	[18]
Vodobatinib (K0706)	BCR-ABL1	Safety, tolerability, pharmacokinetics, and anti-leukemic activity of vodobatinib in treatment-refractory/intolerant CML	Chronic phase	NCT02629692	I/II	[21]
PF-114	Abl1, STAMP	Tolerability, safety, pharmacokinetics, and preliminary efficacy of PF-114 in patients with Ph ​+ ​CML, resistant to 2G TKIs or T315I	Chronic phase	NCT02885766	I/II	[24]
Rebastinib (DCC-2036)	ABL1, SRC, TIE2 or FLT-3	Safety and preliminary efficacy of rebastinib in patients with Ph ​+ ​CML (T315I mutation)	Chronic and acute phase	NCT00827138	I	[26]
Danusertib (PHA-739358)	BCR-ABL1	T315I mutation	Blast phase	NCT00335868	II	[27]
AT9283	BCR-ABL1	Safety of escalating doses of AT9283 in patients with different types of leukemia	All	NCT01431664, NCT00522990,	I/II	[28]
MK0457	BCR-ABL1	T315I mutation	Chronic phase	NCT00405054	II	N/A

2G, second generation; TKI, tyrosine kinase inhibitor; CML, chronic myeloid leukemia.

### 2.1 Flumatinib

Flumatinib is a novel oral 2G TKI that has shown substantial safety and efficacy in the treatment of chronic phase (CP)-CML [16]. Flumatininb responses were superior over Imatinib, with a similar safety profile; therefore, it is recommended as a frontline treatment option [17]. Currently, phase VI clinical trials of flumatinib as monotherapy have shown remarkable results in patients with newly diagnosed Ph^+^ CP-CML.

### 2.2 Olverembatinib

Olverembatinib (HQP1351) is a contemporary oral 3G TKI that has shown high efficacy in patients with TKI-resistant CML, including those with the T315I mutation, in a phase II trial [18]. HQP1351 has a low affinity for other kinases, as evidenced by the findings of a phase I trial, in which 63 ​% of patients with CML failed to respond to other TKI therapies and acquired the T315I mutation [19]. Patients with CP-CML achieved 95 ​% complete hematologic response (CHR), 69 ​% major cytogenetic response (MCyR), 61 ​% complete cytogenetic response (CCyR), and 37 ​% major molecular response (MMR) after 12 months of follow-up. A phase II clinical trial of HQP1351 in combination with decitabine for advanced CML is currently underway**.** Similarly, several trials assessing the pharmacokinetics, efficacy, and safety of HQP1351 are in progress (Table 1).

### 2.3 Vodobatinib

Vodobatinib (K0706), another novel oral 3G TKI, has significant activity against BCR-ABL mutations, excluding T315I, *in vitro* [20]. Vodobatinib showed an ample safety profile in a phase I trial involving patients with CML who failed to respond to other TKIs [21]. A phase I/II clinical trial assessing the safety and anti-leukemic potential of vodobatinib in patients with Ph^+^ CML who are resistant/intolerant to ≥3 prior CML therapies is in progress (Table 1).

### 2.4 PF-114

PF-114 is a novel orally bioavailable 4G TKI that acts as an antagonist of the BCR-ABL1 kinase domain and an inhibitor of STAMP. It also suppresses the integral stimulation of phosphatidylinositol-3 kinase (PI3K)/AKT/extracellular signal-regulated kinase 1/2 (ERK1/2) or Janus kinases (JAK)/signal transducer and activator of transcription proteins 3/5 (STAT3/5) signaling and increases p27 levels, which causes G1 cell cycle arrest [22,23]. PF-114-induced apoptosis has been observed in patient-derived KCL22 and K562 ​cells. A phase I/II trial indicated the achievement of MCyR and MMR in 55 ​% and 36 ​% of patients, respectively, receiving 300 ​mg of PF-114 [24] (Table 1).

### 2.5 Rebastinib

The recently discovered potent BCR-ABL1 inhibitor, rebastinib (DCC-2036), has a broad spectrum of activity against kinases, including Abl1 kinase, Src kinase, TIE2, and FLT-3, but no activity against KIT. It has the potential to inhibit various BCR-ABL1-resistant mutants including the T315I mutant [25]. The pharmacokinetics of DCC-2036 in patients with Ph^+^ CML with the T315I mutation are unknown, and the current status of the only multicenter phase I clinical trial of DCC-2036 is also unknown (Table 1).

## 3 Signaling pathways in CMLSCs

**Fig. 1:**
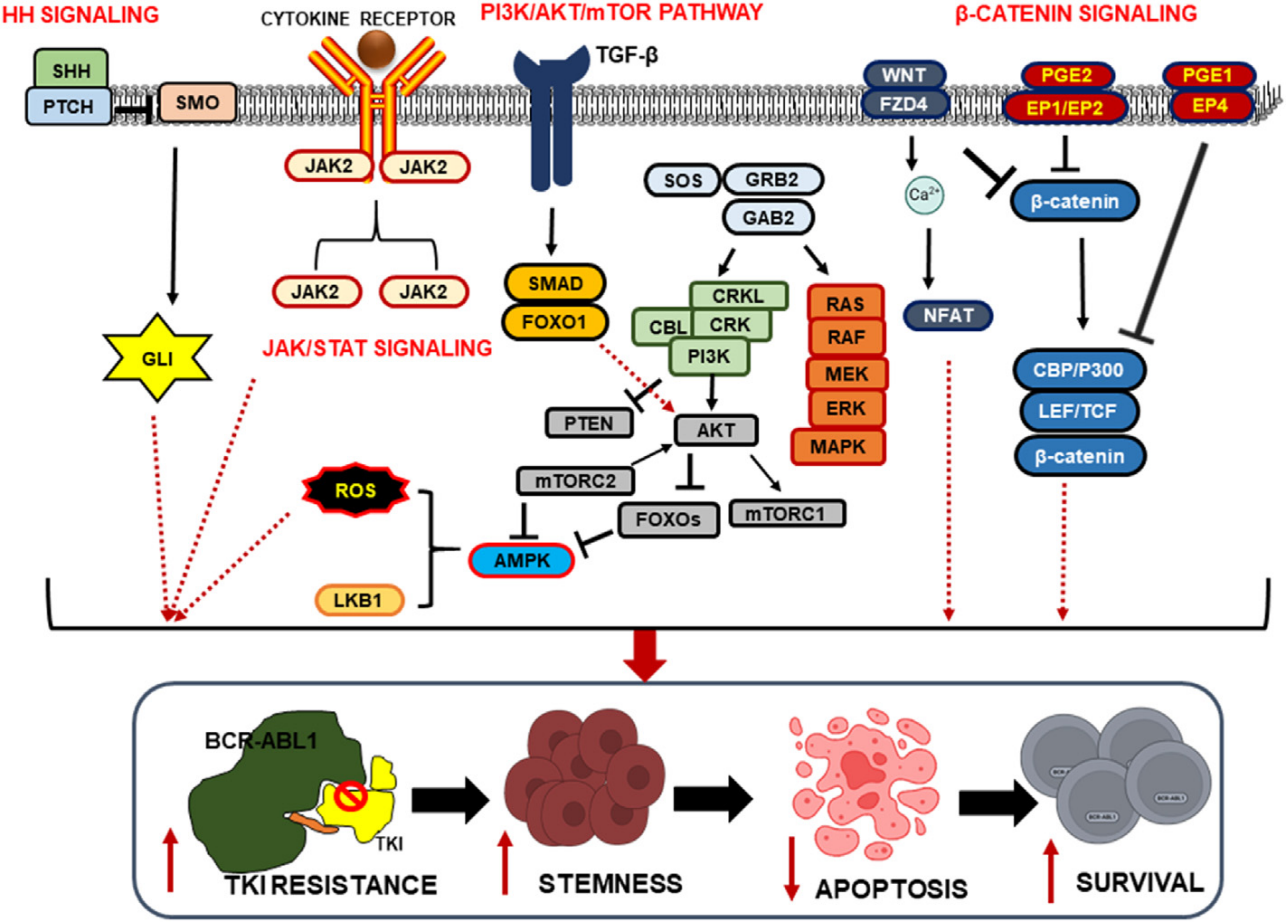
**Signaling pathways involved in CML-LSC survival, stemness, and TKI resistance.** The Hedgehog (Hh) pathway involves the binding of the PTCH receptor with its ligand, sonic hedgehog (SHH) thereby inhibiting smoothened (SMO) protein, leading to the expression of glioma-linked oncogene (GLI), which in turn inhibits apoptosis and promotes leukemia stem cell (LSC) survival. JAK/STAT signaling activation increases the expression levels of anti-apoptotic genes and pro-survival genes. The GRB2/GAB2/SOS complex causes constitutive activation of the downstream RAS pathway, thereby activating MEK1/2 and MAPK proteins and resulting in abnormal cell proliferation and tyrosine kinase inhibitor (TKI) resistance. In addition, this complex also activates the PI3K/AKT pathway. Moreover, AKT activation upregulates mTOR (mTORC1 and mTORC2), resulting in overproduction of reactive oxygen species (ROS) and mitochondrial dysfunction. Chronic myeloid leukemia (CML)-LSCs may also uniquely depend on WNT/β-catenin signaling for survival in the context of BCR-ABL1 kinase inhibition.

Three main signaling pathways are responsible for constitutive BCR-ABL1 activation in CMLSCs: the signal transducer and activator of transcription 5 (STAT5), Ras/mitogen-activated protein kinase (MAPK), and PI3K/AKT pathways. In addition to these pathways, the WNT, FOXO, Hedgehog (Hh), and JAK2/STAT3 signaling pathways contribute to the survival or maintenance of CMLSCs [8,29] (Fig. 1). Upregulation of these signaling pathways causes elevated genomic instability and reactive oxygen species (ROS) levels, leading to the malignant progression of CML [30].

### 3.1 Hh signaling

Hh signaling is involved in cell proliferation, stress/injury-induced responses, and stem cell maintenance. This is accomplished via sonic hedgehog (SHH, Hh ligand) binding to the patched Hh receptor (PTCH), leading to the activation of smoothened (SMO) and glioma-associated oncogene (Gli) transcription factors for downstream signaling (BCL-2, Cyclin D1, MYC, SOX2) [31]. Vismodegib, an inhibitor of Hh, induces growth inhibition and apoptosis of CML cell lines, and its combination with dasatinib significantly enhances cytotoxicity [32]. Sonidegib (LDE225) is a potent and highly selective SMO inhibitor that effectively eradicates CMLSCs, either alone or in combination with TKIs. This is further evidenced by a reduced number of LSCs and improved relapse-free survival following SMO inhibition [32]. In addition, the combination of sonidegib and nilotinib decreases the colony-forming capacity of CD34^+^ cells from patients with CP-CML [33].

### 3.2 PI3K/AKT/mTOR pathway

The PI3K/AKT/mTOR pathway is considered to be the center of oncogenic signaling, downstream of various receptor and growth factor signaling pathways [34]. ROS accumulation, caused by the dysregulation of PI3K/AKT/mTOR signaling, promotes the proliferation, stemness, and survival of LSCs [35]. A preclinical study showed that metformin (an AMPK activator) blocked the PI3K/AKT/mTOR pathway and led to the apoptosis of LSCs [36]. The combination of metformin and TKIs has a synergistic effect on promoting TKI-mediated apoptosis [37].

### 3.3 JAK/STAT signaling

During normal hematopoiesis, the intracellular TK, Janus kinase (JAK2), is activated following the binding of hematopoietic growth factors to their receptors. JAK2 subsequently phosphorylates STAT5, leading to its nuclear translocation. Nuclear STAT5 regulates the transcription of genes involved in normal hematopoiesis [38]. In CML cells, JAK2 is constitutively active and directly binds to the SH2 domain of BCR-ABL1, leading to uncontrolled STAT3/5 phosphorylation. However, JAK2 also stimulates cytokine-mediated signaling in CML cells. CMLSCs show higher expression levels of the thrombopoietin receptor (MPL), with enhanced proliferative and regenerative abilities [39]. CMLSCs with higher MPL expression levels show increased JAK/STAT signaling, which reduces their sensitivity to TKIs and increases their sensitivity to JAK inhibitors. Therefore, CMLSCs with high MPL expression levels are potential targets for JAK2 inhibitor therapy for LSC depletion in TKI-treated patients [40]. Multiple clinical trials of the ruxolitinib, a JAK2 inhibitor, are in progress, including trials of the combination of ruxolitinib and nilotinib in patients with CML (ClinicalTrials.gov ID: NCT01702064) and Ph^+^ acute lymphocytic leukemia (ClinicalTrials.gov ID: NCT02253277). Hence, combination therapy with JAK2 inhibitors and TKIs may be an effective option for eradicating LSCs [41].

### 3.4 β-Catenin signaling

Nuclear β-catenin is required for the survival and self-renewal of normal HSCs and CMLSCs [42]. C82, a β-catenin inhibitor, eliminates LSCs by downregulating the expression of CRKL, CD44, STAT5, c-MYC, and survivin [43]. Moreover, a combination of C82 and nilotinib eradicates IM-resistant CML cells and prolongs the survival of xenotransplants [44]. Furthermore, the combination of WNT974 (a potent inhibitor of PORCN, which is involved in the acylation of WNT) and nilotinib markedly reduced the number of CMLSCs and other progenitors by suppressing AXIN-2, cyclin-D1, and c-MYC expression *in-vivo* [45,46].

### 3.5 BCL-2

Bcl-2 protects LSCs from drug-induced apoptosis via the PI3K/AKT and JAK/STAT pathways due to its anti-apoptotic potential [47]. In addition to BCL-2, BCR-ABL1 activates other anti-apoptotic proteins, such as B-cell lymphoma-extra-large (BCL-XL) and MCL-1 [48]. The combination of venetolcax (a BCL-2 inhibitor) and TKI synergistically promotes the apoptosis of CD34^+^ CML cells by targeting mitochondrial oxidative phosphorylation [49].

### 3.6 MAPK/MNK1/2 pathway (MNK-eIF4E axis)

The blast-crisis (BC) phase of CML is attributed to higher β-catenin signaling activity in granulocyte macrophage progenitors (GMPs), enabling CML cells to act as LSCs and reservoirs for resistance. Therefore, the MAP kinase interacting serine/threonine kinase (MNK)-eukaryotic translation initiation factor 4E (eIF4E) axis is overexpressed in GMPs of BC phase, but not in normal HSCs [50]. MNK kinase-dependent eIF4E phosphorylation at serine 209 stimulates β-catenin translocation and nuclear activation in BC GMPs, which contributes to TKI resistance and leukemogenesis. Preclinical studies have demonstrated that eIF4E phosphorylation or β-catenin signaling is suppressed by ETC-1907206, a selective inhibitor of MNK1/2 [50].

### 3.7 BCR-ABL1/Gab2/Grb2 axis

Dysregulated expression of growth factor receptor–bound protein 2 (GRB2) allows binding to the SH2 domain of BCR-ABL1, causing the formation of GRB2-SOS complexes and resulting in downstream hyperactivation of the RAS/MAPK pathway. BP1001 is a liposome-incorporated *GRB2*-specific antisense oligonucleotide that inhibits GRB2 expression and RAS/MEK/ERK signaling, while possessing a synergistic effect with TKIs [51]. The combination of trametinib (a MEK inhibitor) and IM restores TKI sensitivity by inhibiting MEK/ERK-mediated LSC survival [52].

### 3.8 PPARγ/STAT5/HIF2α axis

Hypoxia-inducible factor-2α (HIF-2α)/CITED functions as a downstream target of STAT5 activation to maintain the stemness and quiescence of CMLSCs [53]. Given that peroxisome proliferator-activated receptor gamma (PPARγ) activation reduces STAT5 expression levels [54], PPARγ activators, including glitazones and pioglitazone, combined with IM, have been shown to clear residual CMLSCs, as confirmed in phase II clinical trials in patients with CML (ClinicalTrials.gov ID: NCT02888964, NCT02889003) [55]. Thiazolidinediones, as PPARγ agonists, have also been shown to prevent LSC invasion or adhesion by upregulating MMP-9 or MMP-2, and prevent LSC apoptosis via caspase-3 stimulation [55]. Furthermore, PPARα ligands, such as clofibrate and WY-14643, upregulate the expression of human organic cation transporter 1 (hOCT1), leading to greater uptake of imatinib to induce TKI-associated apoptosis [56].

### 3.9 SDF1/CXCR4/CXCR7 axis

Preliminary studies have shown that CXCR4/CXCR7, a CXCL12 receptor, regulates the migration and homing of normal and malignant hematopoietic cells [57]. *In vitro* and *in vivo* studies have revealed that NOX-A12, a novel CXCL12 inhibitor, combined with nilotinib, eliminates CML cells by inhibiting SDF1-mediated migration [58].

## 4 Novel LSC-targeting therapies for CML

**Fig. 2:**
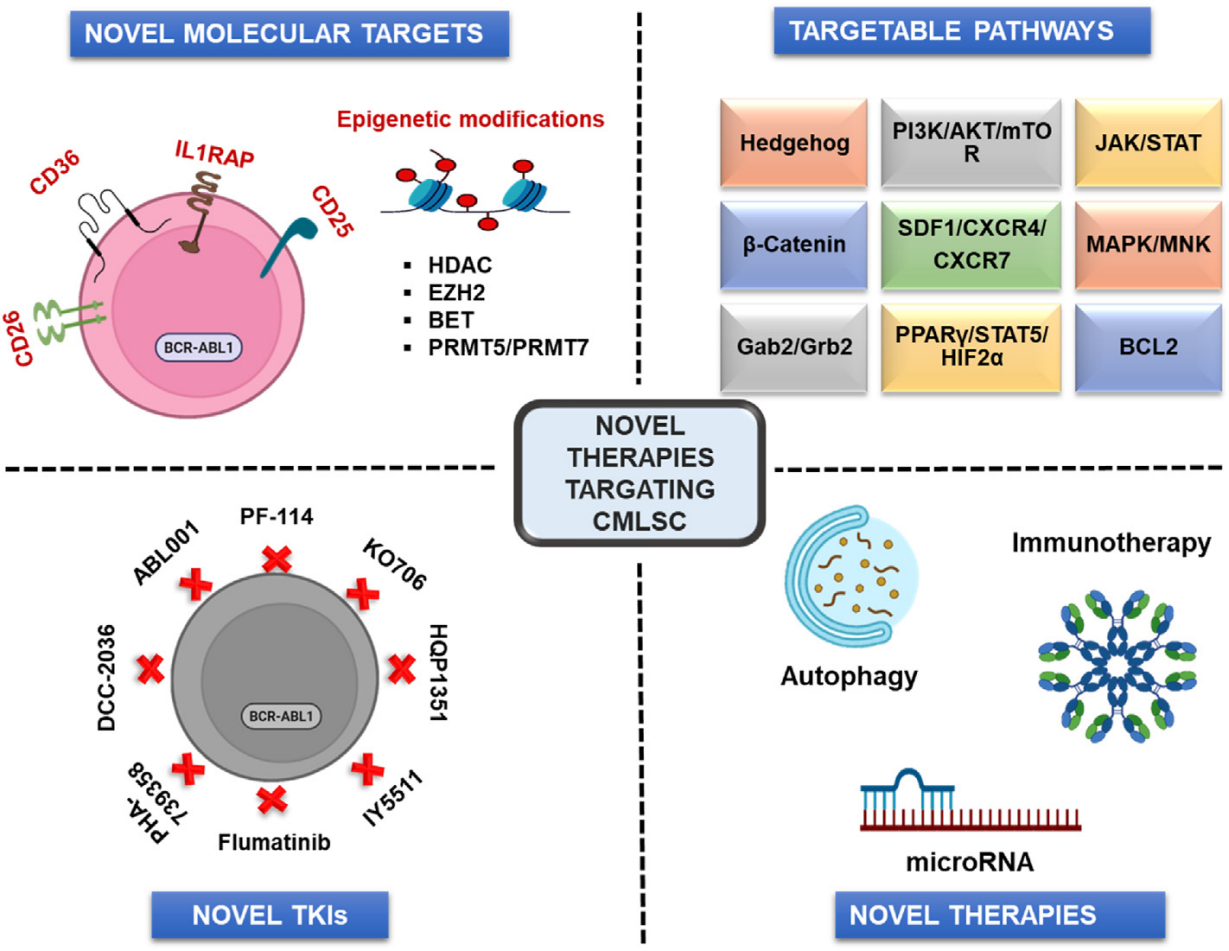
**Novel therapeutic approaches against CML.** Novel therapeutic approaches against chronic myeloid leukemia (CML) include novel molecular biomarkers, signaling pathways, tyrosine kinase inhibitors (TKIs), and targeted strategies.

The eradication of CMLSCs is another critical strategy for treating CML. Therefore, the molecules involved in the stimulation, proliferation, survival, and quiescence of CMLSCs are potential therapeutic targets for the treatment of CML (Fig. 2).

### 4.1 Novel molecular targets in CMLSCs

#### 4.1.1 PEG1-EP4

Prostaglandin E1 (PGE1) and PGE2 are structurally similar. They are 20-carbon fatty acid derivatives generated from the oxygenation of dihomo-γ-linolenic acid. PEG2 enhances CML-LSC activity and TKI resistance via β-catenin activation [59]. In contrast, PGE1 acts on the EP4 receptor and represses the AP-1 transcription factors, FOSB and FOS, in a β-catenin-independent manner [60]. In line with this, the EP4 receptor agonist misoprostol (a PGE1 analog) also impairs CML-LSC activity. Therefore, PGE1-EP4 activation is a potential target for eradicating CMLSCs.

#### 4.1.2 E-selectin

The bone marrow microenvironment (BMM) has a direct impact on LSCs, and their crosstalk is facilitated by cell-cell interactions and soluble or exosome-encapsulated factors. Therefore, direct cell-cell interactions between CMLSCs and components of the BMM are of great importance, because the adhesion of LSCs is responsible for homing and engraftment. Engraftment and adhesion are mediated by the expression of integrins, selectins, and CD44 [61]. For instance, LSC dormancy and TKI resistance have been associated with elevated CD44 expression levels and increased E-selectin binding in bone marrow (BM) endothelial cells [62]. Moreover, interactions between E- and P-selectins and VLA4 mediate HSC rolling and homing, whereas SDF1 and CXCR4 serve as chemo-attractants via β1/2-integrins. BCR-ABL1 upregulates the SDF1/CXCR4 axis in CML, leading to selective homing and survival in the BM niche. CMLSCs modify external factors and upregulate CD44^+^ or E-selectin expression to mediate significant BMM alterations, such as BM fibrosis [63]. GMI-1271 (uproleselan), an E-selectin inhibitor, induces the migration of homed LSCs from the BM niche into the peripheral blood for cellular differentiation. *In vitro* results indicate that cell cycle progression is altered (CDK6 upregulation or *p16* downregulation) by either GMI-1271 monotherapy or combination therapy with IM [63] (Table 2). Preclinical studies aimed at eradicating LSCs in CML are currently underway.

**Table 2:** Novel potential molecular targets of CMLSCs with their inhibitors.

Molecular Target	Inhibitor	Mechanism of Action	ClinicalTrials.gov number	Ref.
E-Selectin	GMI-1271	Migrates homed LSCs from bone marrow niche into peripheral blood	NCT02811822	[63]
Hypoxia-inducible factor	Acriflavine	Stimulates p57 and p1ARF, Downregulates MYC and blocks OCT4 and SOX9	N/A	[93]
MYC	10058-F4	Suppresses CIP2A, leading to PPA2 reactivation	N/A	[94]
SET	OP449 and FTY720	Reactivates PPA2 i*n vitro*	N/A	[95]
MDM2	AMG-232 DS-5272 SIRT1 RITA with CPI-203	Prevents the p53-MDM2 interaction Prevents the p53-HDM-2 interaction Prevents p53-BCR-ABL1 interaction Prevents p53-IκBα interaction	NCT04835584	[70,71,73,74]
CD26	Gliptins/begelomab	Disrupts the SDF1/CXCR4 axis	NCT01720264	[96]
IL1RAP	Anti-IL1RAP antibody	Unknown	NCT02842320	[77]
CD36	CD36-targeting antibodies	Reduces IM sensitivity and CD36-mediated FA transport/uptake/oxidation	NCT04104035	[78]
HDAC	Panobinostat	Prevents the transcription of targeted genes	NCT00451035	[84]
	MAKV-8		NCT02543749	[82]
	Chidamide		N/A	[85]
EZH2	EPZ-6438	Upregulates p16 *in vitro*	NCT04147533	[87]
BET	JQ-1	Downregulate MYC and overcomes TKI resistance	N/A	[88]
	CPI-0610		NCT02158858	[89]
PRMT5	PJ-68	RNA metabolism	N/A	[91]
PRMT7	JS1310	Glycine metabolism	N/A	[92]

IM= Imatinib; FA= Fatty Acid.

#### 4.1.3 Hypoxia-inducible factor

Hypoxia-inducible factor (HIF) guarantees the survival, proliferation, quiescence, and immune evasion of CMLSCs, and TKI resistance in the hypoxic BMM by suppressing BCR-ABL1 [64]. Acriflavine is an HIF-1 inhibitor that causes a reduction in the survival, maintenance, stemness, and formation of LSCs by preventing HIF complex dimerization. These effects of acriflavine occur via three key mechanisms: 1) stimulation of the expression of tumor suppressors, including p57 and p19Arf; 2) inhibition of the expression of c-MYC; and 3) inhibition of the expression of stemness-associated genes, including *OCT4* and *SOX9* [65]. *In vitro* and *in vivo* studies have shown that GMI-1271 is cytotoxic to CMLSCs, with fewer harmful effects on normal HSCs [65] (Table 2).

#### 4.1.4 Protein phosphatase 2A

Protein phosphatase 2A (PP2A) is a tumor suppressor gene that disrupts MYC-MAX interactions by dephosphorylating MYC in CMLSCs. PP2A is inactivated by endogenous inhibitors, such as CIP2A and SET, which are regulated by BCR-ABL1 to maintain LSC quiescence [66]. 10058-F4, a MYC inhibitor, has been shown to suppress CIP2A *in vitro* [67]. OP449 and FTY720 are SET antagonists that reactivate PPA2 *in vitro.* The combination of 10058-F4 and FTY720 displays synergistic effects [68] (Table 2).

#### 4.1.5 TP53 modulation

TP53 plays a vital role in tumor suppression and apoptosis control. Its direct binding to BCR-ABL1 and IκBα makes it dysfunctional in CML, leading to dysregulated proliferation and resistance to apoptosis [69]. The E3 ligase MDM2, a key negative regulator of p53, not only binds to p53 to block its tumor-inhibiting trans-activation domain, but also labels p53 for degradation by the proteasome. Targeting the interaction between p53 and MDM2 represents an attractive treatment approach for cancers with wild-type or functional TP53 [70]. Combined with TKI, DS-5272, an MDM2 inhibitor, reduces the burden of leukemia, while extending survival in mouse models of CML [71,72]. AMG-232, another MDM2 inhibitor, is under clinical trial in patients with CML (ClinicalTrials.gov ID: NCT04835584) (Table 2).

Human double minute 2 (HDM2) is another key negative regulator of p53 expression in CML. MI-219, an HDM2 inhibitor, decreases the engraftment, self-renewal, and homing of LSCs via direct p53 stabilization and reactivation [73], and RITA, a p53-HDM2 inhibitor, in combination with CPI-203, a Bromodomain and Extra-Terminal (BET) inhibitor, decreases LSC engraftment by preventing p53 degradation in a mouse model of CML [74]. DS-5272, an MDM2 inhibitor, also acts as an HDM2 antagonist to restore p53 function. DS-5272, in combination with TKI/BET inhibitors, shows exceptional LSC-eradication activity [71] (Table 2).

#### 4.1.6 CML-LSC surface antigens/biomarkers

CMLSCs are characterized by novel surface antigens or biomarkers with diagnostic, prognostic, and therapeutic significance in the CD34^+^CD38^−^ cell fraction (Table 2). CD26 is a widely expressed LSC surface marker that supports the migration of LSCs from bone marrow into the peripheral blood by cleaving SDF1/CXCR4 axis [11]. Gliptins, CD26 inhibitors, restore the cleaved SDF1/CXCR4 axis to promote LSC homing. Moreover, the combination of venetoclax (a BCL-2 inhibitor) and begelomab (a CD26 antibody) inhibits cell growth, induces the apoptosis of CD26^+^ CMLSCs, and synergistically promotes their function [11]. Similarly, IL1RAP is expressed on surface of all CD34^+^CD38^−^ BCR-ABL1^+^ cells [75]. Chimeric antigen receptor (CAR) T-cell-based immunotherapy, using IL1RAP as a tumor-associated antigen, has been proposed as a novel approach for the treatment of CML [76]. Targeting IL1RAP with anti-IL1RAP antibodies kills CML cells and improves the survival of mouse xenografts by inhibiting IL-1B signaling and inducing antibody-dependent cytotoxicity [77]. Moreover, RNA sequencing analysis revealed that CD36 is markedly upregulated in primitive CML cells with decreased imatinib sensitivity. After dividing CD34^+^CD38^−^IL1RAP^+^ cells into two distinct subgroups based on the expression of CD36, compared to CD36^−^ cells, CD36^+^ cells were found to be highly quiescent or insensitive to IM [78]. A previous study reported that CD36-mediated fatty acid (FA) transport in CMLSCs was reduced by the integrin-linked kinase (ILK) inhibitor dasatinib [44]. In addition, CD36 mediates FA uptake and oxidation in BC LSCs [79]. Furthermore, a high expression level of CD25 inhibits the proliferation of CMLSCs and prevents disease progression. The IL2-CD25 axis actively participates in protecting CMLSCs by promoting LSC proliferation, leading to TKI resistance [80]. The expression of CD25 in CMLSCs is the result of BCR-ABL1-mediated activation of STAT5, and CD25 is upregulated only in LSCs and not in other progenitor cells (e.g., CD34^+^CD38^+^ cells). Of note, CD25 inhibits the growth of other progenitor CML cells. Therefore, the discovery of drugs that can stimulate the re-expression of CD25 will enable the clearance of CM-LSCs. BEZ235 (PI3K and mTOR inhibitor) and rapamycin (an mTOR inhibitor) have been reported to induce CD25 re-expression [81].

### 4.2 Targeting epigenetic modifications

Epigenetic modification is an important way to maintain LSCs, independently of BCR-ABL1 kinase activity. Targeting epigenetic regulators is an important strategy for eliminating LSCs in CML (Table 2).

#### 4.2.1 Histone deacetylase

Overexpression of histone deacetylase (HDAC) facilitates the acetylation of lysine on tightly packed histones to prevent the transcription of specific genes, including tumor-suppressor genes [82]. The combination of IM and HDAC inhibitors (HDAC-Is) successfully and efficiently targets quiescent CMLSCs [83]. Panobinostat (LBH589), a potent HDAC-I, combined with TKIs, promotes TKI-mediated apoptosis and disrupts LSC quiescence via Hsp90 acetylation, with enhanced proteasomal degradation of key CM-LSC signaling proteins. Panobinostat monotherapy also inhibits the growth of various CML cell lines, even those with the T315I mutation [84]. Chidamide is another HDAC-I that induces apoptosis by increasing histone H3 acetylation and caspase3/9 activation and decreasing β-catenin levels [85]. Moreover, MAKV-8 is a novel pan-HDAC-I that eradicates LSCs by reducing c-MYC expression levels and caspase3/9 activation and triggering endoplasmic reticulum stress *in vitro* [82].

#### 4.2.2 Enhancer of zester homolog 2

Enhancer of zester homolog 2 (EZH2), a member of the polycomb repressive complex 2 (PRC2) and histone methyltransferase family, induces histone H3 methylation and inactivates transcription. The survival or expansion of CM-LSCs and TKI resistance are mediated by EZH2 hyperactivity, which blocks myeloid differentiation [11]. EZH2 inhibition alters epigenetic reprogramming, leading to the increased sensitivity of CMLSCs to apoptosis, and the combination of an EZH2 inhibitor and a TKI can effectively eradicate LSCs [86]. Similarly, EPZ-6438 (an EZH2 inhibitor) induces upregulation of the *p16* tumor suppressor gene *in vitro* to eradicate leukemic cells [87].

#### 4.2.3 Bromodomain and extra-terminal proteins

Bromodomain and extra-terminal (BET) proteins are epigenetic, transcriptional, inflammatory, and cell-cycle regulators. Bromodomain-containing protein 4 (BRD4) activation and MYC upregulation are driven by LSCs and BCR-ABL1 acquired senescence-associated secretory phenotype (SASP) causing release of pro-inflammatory cytokines to facilitate LSC senescence. JQ-1, a potent BRD4 inhibitor, downregulates MYC and overcomes TKI resistance. dBET6 and dBET1 are two potent BRD4 inhibitors that eliminate CD34^+^/CD38^-^ LSCs [88]. Additionally, CPI-0610, a novel BRD4 inhibitor, is currently undergoing phase I clinical trials (ClinicalTrials.gov ID: NCT02158858) [89].

#### 4.2.4 Protein arginine methyltransferases

Arginine N-methylation is catalyzed by a class of enzymes known as protein arginine methyltransferases (PRMTs) that covalently link methyl groups to arginine side chains [90]. PRMT5, a type II arginine methyltransferase, is required for the survival and self-renewal of CMLSCs. Mechanistically, PRMT5 regulates DVL3 to increase β-catenin levels. PJ-68, a selective inhibitor of PRMT5, significantly decreases the self-renewal capacity of CMLSCs *in vitro* and *in vivo* [91]. PRMT7, the only type III arginine methyltransferase, reprograms glycine metabolism to promote the detoxification of CMLSCs, allowing for their persistence. JS1310, a specific inhibitor of PRMT7, eliminates LSCs without affecting normal hematopoiesis [92].

### 4.3 Targeting altered metabolism

Targeting the altered metabolic processes in CML cells is a potential therapeutic approach. Human and murine CML models have revealed a direct relationship between branched-chain amino acid transferase (BCAT1) levels and disease progression [97]. CML stemness and amino acid signaling are regulated by dipeptides that are highly expressed in CMLSCs via activation of the SMAD3 and p38MAPK pathways. The inhibition of dipeptide uptake by targeting SMAD3 signaling results in impaired CML-LSC activity [98].

Sirtuin 1 (SIRT1) is a potent p53 suppressor and NAD^+^-dependent histone deacetylase that compensates for the bioenergetic requirements of LSCs [99]. SIRT1 or downstream elements of the oxidative phosphorylation pathway are potential therapeutic targets of SR-18292 (PGC-1α inhibitor), alone or in combination with TKIs, to prevent CML relapse. Additionally, SIRT1 inhibition induces p53-mediated CML-LSC apoptosis, while the SIRT inhibitor/TKI combination reduces TKI resistance and suppresses CML progression [97].

### 4.4 Targeting autophagy

Autophagy is an important pathway for the resistance of BCR-ABL1^+^ cells to IM. The inhibition of autophagy, in combination with TKIs, has the therapeutic potential to eradicate LSCs, and targeting autophagy-related genes leads to primary CML cell death [100]. For example, spautin (an autophagy inhibitor) combined with IM inhibits the PI3K/AKT pathway and downregulates anti-apoptotic genes, leading to the apoptosis of CMLSCs [101]. In addition, chloroquine enhances the sensitivity of CD34^+^/CD38^-^ cells to TKI-mediated apoptosis [102]. Furthermore, knockout of ATG4B, ATG5, or ATG7 inhibits the survival of CMLSCs and increased their susceptibility to TKIs [103,104].

### 4.5 Immunotherapy

The combination of pegylated interferon-alfa (PEG-IFN-α) and TKIs has been shown to be an effective therapy for patients with newly diagnosed CML, but overcoming toxicity remains challenging [105]. GVAX therapy (administration of *ex-vivo*-modified tumor cells) [106] and immune checkpoint inhibition are also important new therapies for CML. Programmed death-1 (PD-1) receptor and its ligand (PD-L1) eradicate CMLSCs by mediating self-tolerance and PD-1 inhibition through T-cell immunotherapy [107].

### 4.6 MicroRNA targeting

Leukemogenesis and TKI resistance are also caused by microRNA dysfunction [108]. For example, overexpression of *miR-29a* causes a reduction in TET2 or EPAS1 levels and the upregulation of BCL-2 or MCL-1. In addition, dysregulation of the c-MYC/*miR-150* axis leads to the downregulation of *miR-153-3p* (a BCL-2 inhibitor) and impaired TKI resistance and myeloid differentiation of CMLSCs. Moreover, decreased expression levels of *miR-424* or *miR-142* and overexpression of *miR-217* result in the release of excessive amounts of oncoproteins.

### 4.7 Protein synthesis

Omacetaxine is a semi-synthetic subcutaneous protein synthesis inhibitor approved for patients with CP- and acute-phase-CML who develop resistance to two or more TKIs. However, it is not frequently used because of its inadequate levels of myelosuppression and inconvenient administration [109]. Of note, 77 ​% of patients with CML show complete hematologic response (CHR) when treated with omacetaxine, which leads to CML-LSC apoptosis [110]. Two multicenter trials of macetaxine are ongoing for patients with CML who have developed resistance to at least two TKIs (ClinicalTrials.gov ID: NCT00462943 and NCT02078960).

## 5 Conclusions

In this review, we provide a deeper understanding of CML at the molecular level by examining targeted therapies for CML. Previous studies have focused on tyrosine kinase inhibitors and their combination therapies to improve the survival of patients with CML. Targeting LSCs also has significant therapeutic potential in CML. To overcome TKI resistance and eliminate the LSCs that mediate CML progression, new drug targets that focus on epigenetic modifications and the BM microenvironment and related signaling pathways are emerging. There is an increasing number of reports related to CML-targeted therapy; however, there are still some major challenges with such therapies, such as TKI resistance, TKI toxicity, and LSC resting states, which require the development of new therapeutic strategies. Targeting BCR-ABL1 degradation using other molecular pathways may be a promising approach for eradicating LSCs. Additionally, TKI resistance can be overcome by exploring combinations that synergistically target multiple pathways.

## Funding

This work was supported by the National Natural Science Foundation of China (grant number 81874294); Shandong Province (grant number TSQN201812015), the Shandong University (grant number 2020QNQT007); and the Natural Science Foundation of Shandong Province (grant number ZR2022LSW027).

## CRediT authorship contribution statement

**Muhammad Sameer Ashaq:** Writing – original draft. **Qian Zhou:** Writing – original draft. **Zhuoran Li:** Writing – original draft. **Baobing Zhao:** Writing – review & editing.

## Declaration of competing interest

The authors declare that they have no known competing financial interests or personal relationships that could have appeared to influence the work reported in this paper.
